# Supplementary material for: Cultural conformity generates extremely stable traditions in bird song
Source: Nat Commun. 2018 Jun 20;9:2417. doi: 10.1038/s41467-018-04728-1 (PMC6010409; doi:10.1038/s41467-018-04728-1)

Supplementary Information

"Cultural conformity generates extremely stable traditions in bird song"

Lachlan et al.

Supplementary Note 1 Analysis of variance in syllable type attractiveness

We reanalysed playback data from Lachlan et al. 2014. In this study, versions of 9 different common syllable types were played back to a total of 68 territorial male swamp sparrows. While these males discriminated between well-learned and poorly learned versions of these syllable types, there was little effect of syllable type itself. In the original study, syllable type was modelled as a random effect. We reanalysed the data using a linear model, treating syllable type as a fixed effect, and found no evidence that it influenced male response (effect of including factor syllable type: delta AIC=4.04, df=8, F=1.394, p=0.21). This justifies our model, in which content biases divided syllable types into "attractive" and "unattractive" categories, and set the inherent attractiveness of syllable types within former category equal.

Supplementary Note 2: The lack of a trade-off between mutation rate and population size in simulations.

Based on classical population genetics, one might expect a trade-off between mutation rate and population size. The ramifications of such a trade-off would be a given pattern of population diversity could be generated by a wide range of mutation rates, each with a corresponding population size. Given the broad priors we chose for population size, this trade-off would be expected to limit our ability to infer mutation rate, yet in fact the posterior distribution of mutation rate was fairly narrow (Fig. S3E).

To examine why, we carried out two further sets of simulations. In one set of simulations, we set parameter settings of v=0.01, p_{att}=1, N_T=5 and α=1.33. In the other set of simulations, we set α=1.0 (i.e. no conformist bias). We drew 1000 values of μ and N_P from prior distributions (log-uniform, limits 0.001, 0.1 for μ and 400, 3000 for N_P) and ran simulations for each set (sampling 206 individuals from each simulation, as for the Conneaut population). We then measured the deviation of each simulation run from each other simulation run using our PLS dimensions, as described in the Methods. We then selected the 0.1% of comparisons with the smallest deviation from another. For each of these comparisons, we measured the ratio of their mutation rates (μ-ratio) and the ratio of their population sizes (N_P-ratio). These ratios might be thought of as the ability of the simulations to estimate these parameter values: if they cluster around 1, it suggests a good ability to predict the parameter.

According to neutral theory we would predict a clear negative correlation between the μ-ratio and the N_P-ratio. This is exactly what we observed in the simulation set with α=1.0 (r=-0.883) (Fig. S7A), but when we included a conformist bias (α=1.33), this correlation was much weaker (r=-0.300) (Fig. S7B). The addition of the conformist bias changed the relationship between mutation rate, population size and diversity such that a reliable estimate of mutation rate could be achieved.

Supplementary Table 1 Demographic and sampling details of populations

Details of the six populations studied. 1. Populations differed in their isolation from other population. ‘High’ levels of isolation means that no population of equivalent size is found within 30km; ‘Medium’ means that similar-sized populations are found occasionally within 30km; ‘Low’ means that the studied population is embedded in a much larger region of clearly inter-connected populations. 2. Populations differed in the degree to which they were fragmented. A ‘High’ level of fragmentation means that there was >10km between population fragments; ‘Medium’ means that population fragments were separated by 1-10km; ‘Low’ means that the populations formed one contiguous area. 3. Populations differed in their recent history. For three, we did not find any records suggesting major changes. For Montezuma Marsh and Horicon Marsh, there is a record of anthropogenic changes (draining and flooding, respectively) that would have been very likely to have temporarily, but greatly reduced the population in size, in recent history (between 1910 and 1935, and between 1846 and 1869, respectively). 4. Population areas were estimated based on a combination of direct examination and inspection of satellite images. 5. The number of territories were estimated based on an extrapolation of the density of birds found in the sampled parts of the population to the entire population area.

**Location**	**State**	**Latitude & Longitude**	**Isolation from other populations[1]**	**Fragmentation of population[2]**	**Population history[3]**	**Population area (km^2)[4]**	**Estimated number of territories[5]**	**Sample Size**	**Mean Repertoire Size**
Hudson Valley (Red Hook)	NY	42.039°N, 73.920°W	Medium	High	Stable	2.66	500	101	3.12
Adirondacks (Raquette Lake)	NY	43.808°N, 74.635°W	Low	Medium	Stable	5.72	1000	70	3.04
Montezuma Marsh	NY	42.982°N, 76.766°W	High	Low	Bottleneck	7.54	1300	71	3.01
Conneaut Marsh	PA	41.586°N, 80.243°W	High	Low	Population reduction	4.39	750	208	3.16
Waterloo	MI	42.361°N, 84.179°W	Low	Medium	Stable	11.74	2000	74	3.04
Horicon Marsh	WI	43.575°N, 88.649°W	High	Low	Bottleneck	43.20	3000	91	2.95

Supplementary Table 2 Loadings for the PLS Components used in the ABC Analysis.

**Summary Statistic**	**Comp 1**	**Comp 2**	**Comp 3**	**Comp 4**	**Comp 5**	**Comp 6**
Prop. Singletons	0.403785836	-0.180468432	-0.023540769	0.208468453	0.034216949	-0.02412686
Prop. Rare types	0.247477402	-0.323033194	-0.404573521	-0.222584303	-0.093465543	-0.420836125
Prop. Intermediate types	-0.14767088	-0.265998118	0.342799433	-0.36677118	-0.089837615	0.025821626
Prop. Common types	0.044286636	0.279115173	-0.255678038	-0.026620355	0.359371738	-0.363079937
Number of syllable types	0.137234094	-0.339359508	-0.248071972	0.189412629	0.323715982	0.191474066
Freq. Commonest type	0.196389134	0.40041912	0.307574469	0.177062031	0.442720771	-0.169183864
H	0.021911002	0.262541161	-0.403112395	-0.405956506	0.113927932	0.187707121
α_p	0.390606462	-0.201175434	-0.159474965	0.199648138	0.200029943	0.15586505
KS statistic for power law	-0.451469109	0.060823085	-0.485269929	0.192137365	0.018619345	0.393871914
Power law discrepancy, n=2	0.556234139	0.156713286	0.02266435	-0.154581033	-0.395636931	0.424520911
Prop. pairs sharing 1 syllable	0.105375987	0.304561081	-0.220181083	-0.339278729	-0.182923132	-0.248624723
Prop. sharing >1 syllable	0.130538604	0.360152672	0.07189544	-0.074447947	0.166756838	0.352013573
$\bar{r}$	0.011106329	0.280704039	-0.138421539	0.56428238	-0.530042738	-0.209587167

Supplementary Table 3 Correlation matrix between posterior distributions of parameters of model 1.

	v	P_{attr}	α	N_T	μ	N_s
P_{attr}	0.093263766					
α	-0.199199563	-0.17947828				
N_T	0.237595972	0.16921057	**-0.69977445**	-		
μ	0.314012224	**-0.6117945**	-0.19507685	0.32965362	-	
N_s	-0.03978835	0.01838526	-0.01864406	-0.07822889	-0.2624858	-
$\overline{N_p}$	0.007771203	-0.00950115	0.04125133	-0.02762346	-0.2015044	0.006801241

Supplementary Figures

Supplementary Fig. 1 Maps of recording areas and territory locations. **A** Locations of the six populations sampled for the study in North-Eastern USA. **B-G** Locations of individual territories sampled within each of the six populations (Hudson Valley, NY; Adirondacks, NY; Montezuma, NY; Conneaut, PA; Waterloo, MI; and Horicon, WI). Maps created using ggplot (D. Kahle and H. Wickham. ggmap: Spatial Visualization with ggplot2. The R Journal, 5(1),144-161. URL http://journal.r-project.org/archive/2013-1/kahle-wickham.pdf), using data from Google maps.

1A

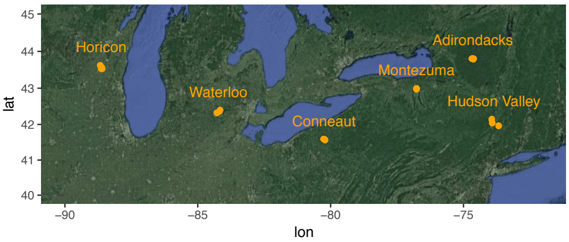

1B

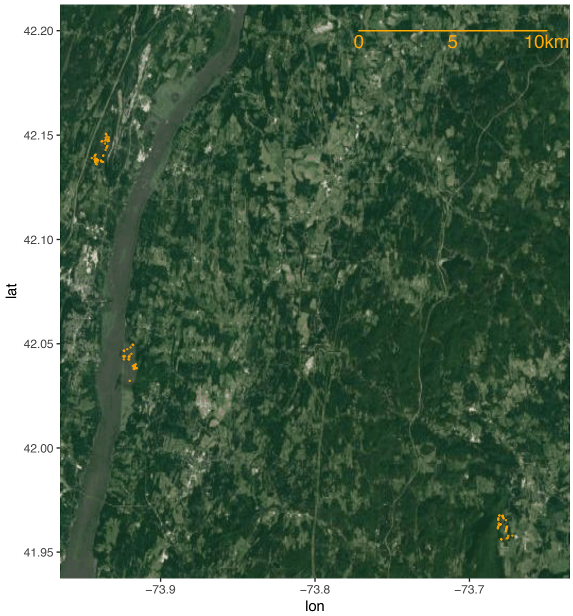

1C

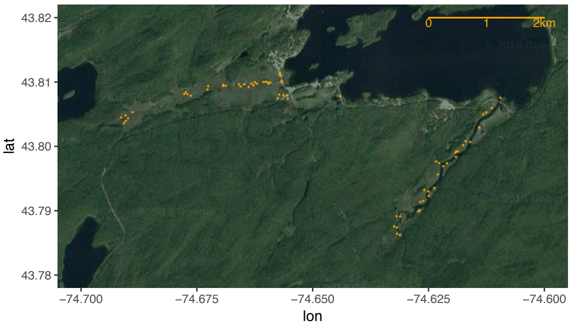

1D

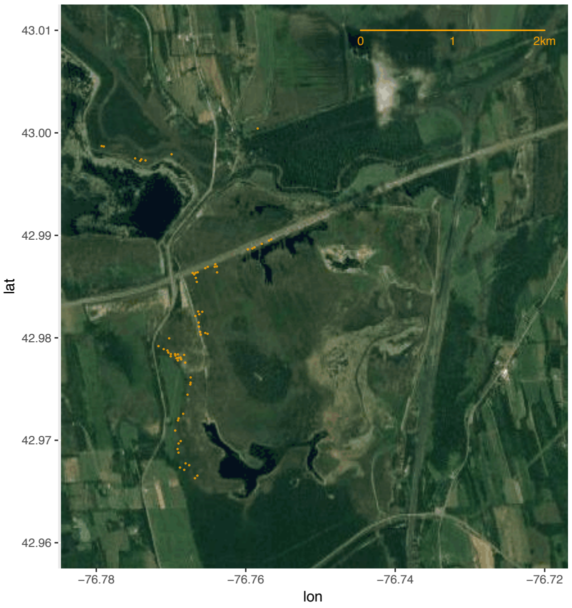
0
1
2km
43.01
43.00
42.99
42.98
42.97
42.96
lat
−76.78
−76.76
−76.74
−76.72
lon

1E

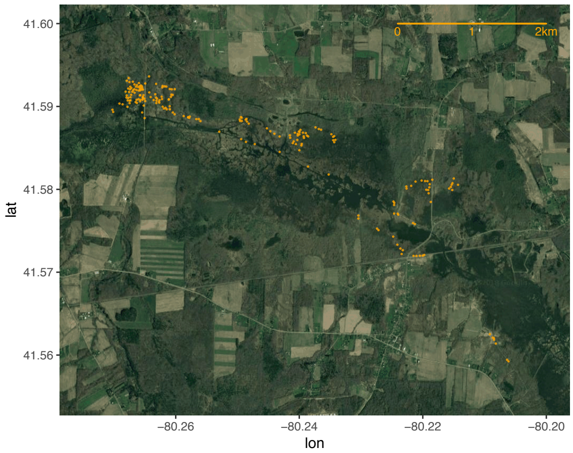
0
1
2km
41.60
41.59
41.58
41.57
41.56
lat
−80.26
−80.24
−80.22
−80.20
lon

1F

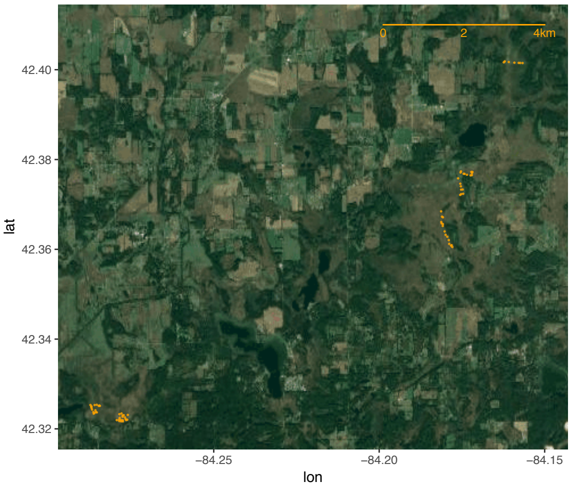
0
2
4km
42.40
42.38
42.36
42.34
42.32
lat
−84.25
−84.20
−84.15
lon

1G

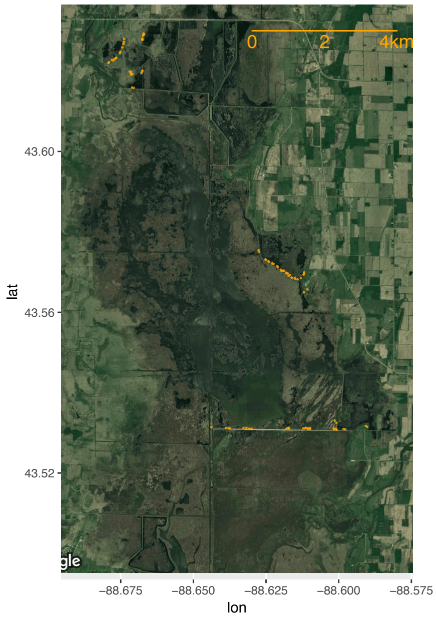
0
2
4km
43.60
43.56
43.52
lat
−88.675
−88.650
−88.625
−88.600
−88.575
lon

Supplementary Fig. 2 Global Silhouette Index (GSI) for the UPGMA dendrogram constructed for syllable types for all 6 populations. A peak in GSI represents evidence for a natural clustering within the data.

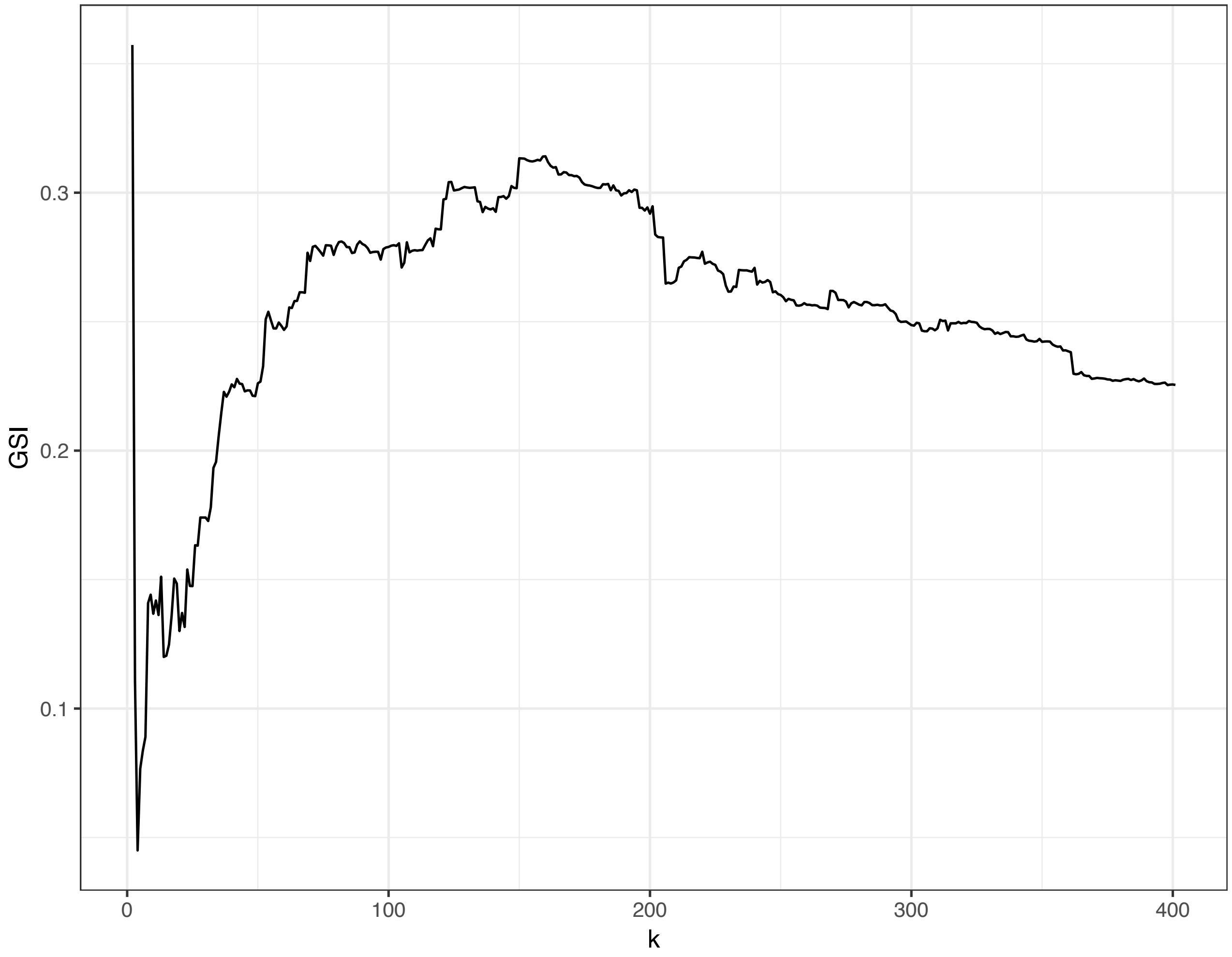

Supplementary Fig. 3 Prior and Posterior probability distributions estimated by ABC for the parameters in the model. Figures show the median (dashed line), the 95% credible interval (blue), and the prior distribution (shaded grey). **A:** v (Model bias). **B:** $\boldsymbol{p_{att}}$ **(**Content bias). **C:** α (Conformist bias). **D:** $\boldsymbol{N_T}$ (Number of tutors). **E:** μ (Mutation rate).

A

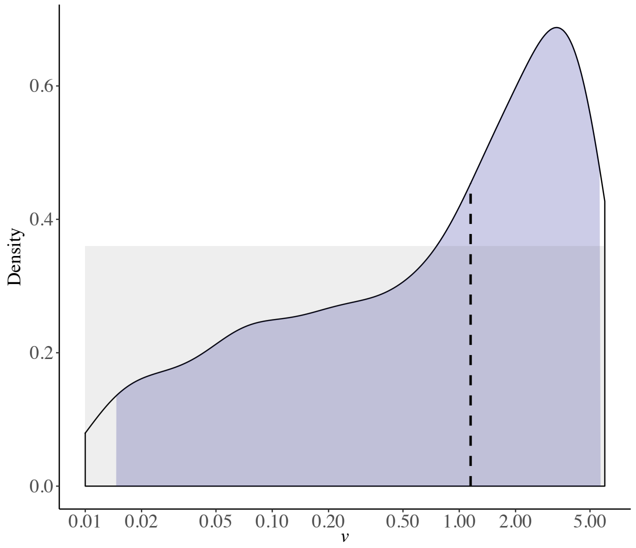

B

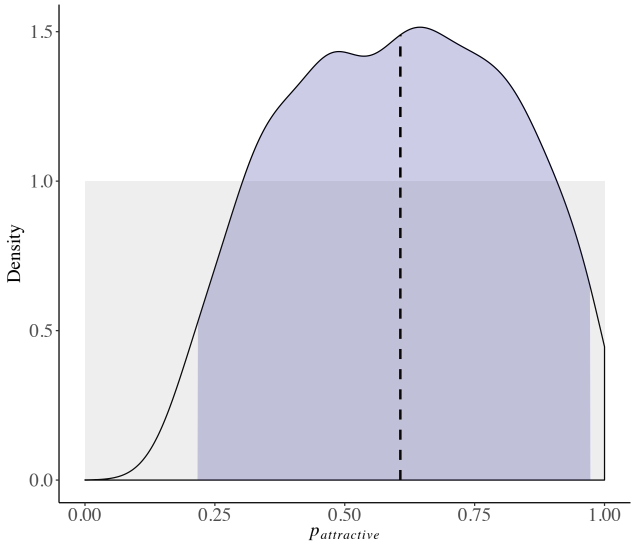

C

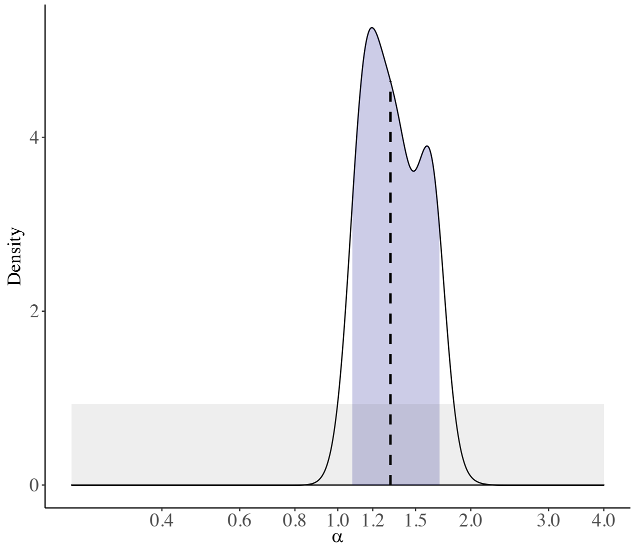

D

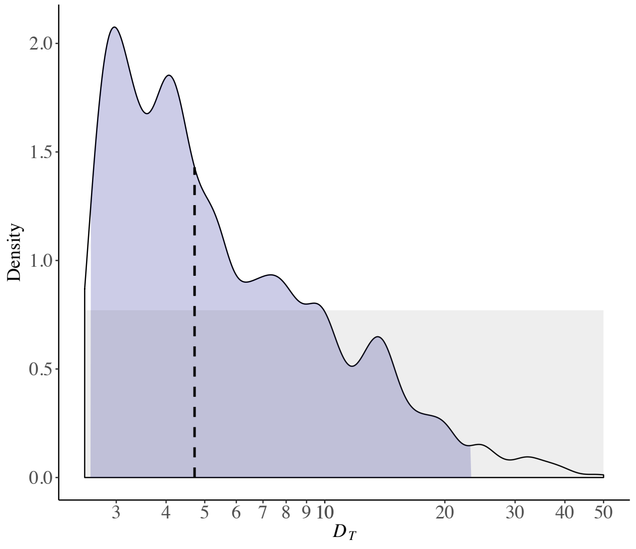

E

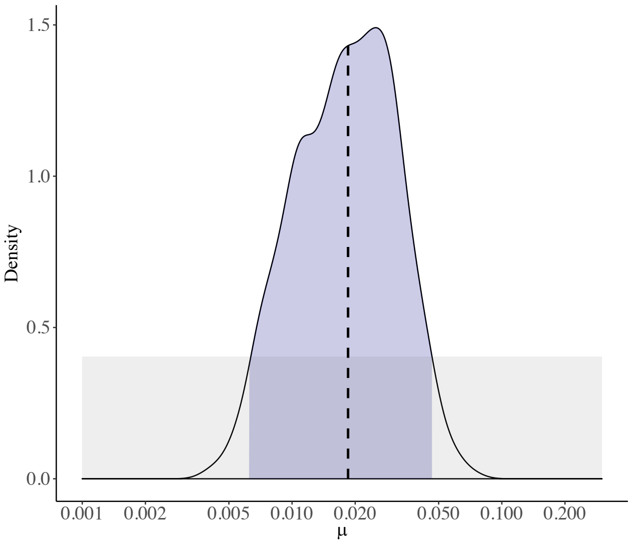

Density
1.5
1.0
0.5
0.0
0.001
0.002
0.005
0.010
0.020
0.050
0.100
0.200
μ

Supplementary Fig. 4 Results of Leave-One-Out cross-validation of the PLS analysis. Y-axis shows the Root Mean Square Error of Prediction for different numbers of PLS components.

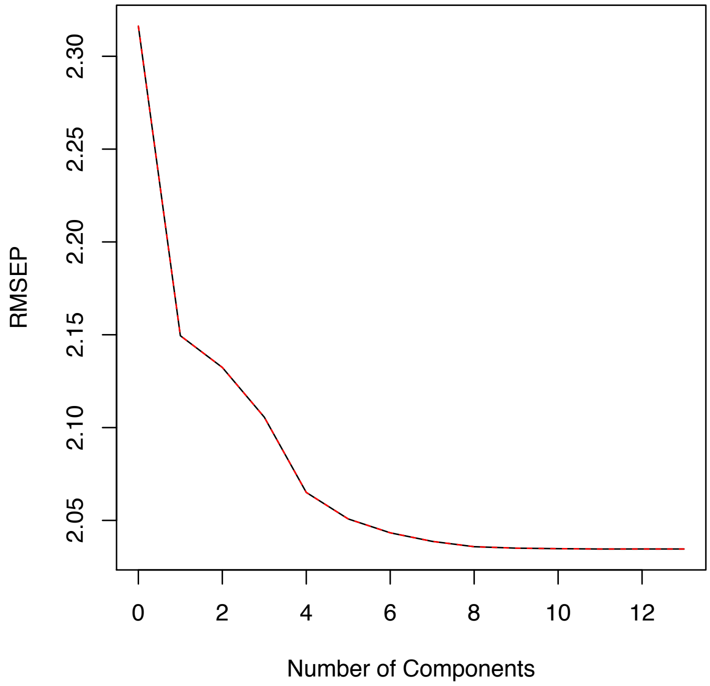

Supplementary Fig. 5 Relationship between model parameters and simulated PLS component summary statistics (s1-s6). The figure illustrates that PLS components clearly covaried with α, μ, and N_T , but not so with N_I in particular.

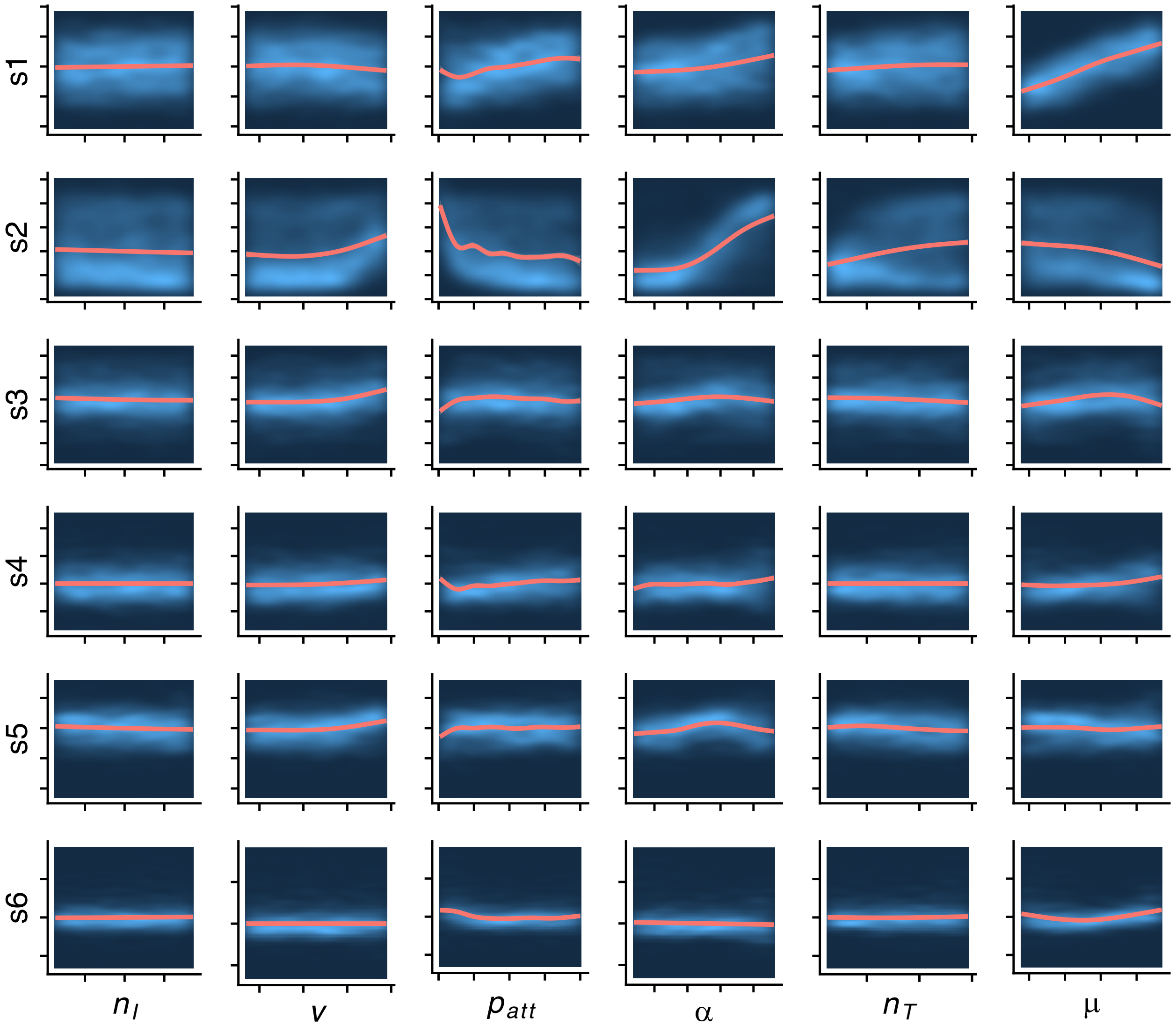

Supplementary Fig. 6 Results of leave-one-out cross-validation analysis. The ability of the ABC procedure to reliably estimate parameter values was examined by taking individual simulation runs, with parameter values sampled from the priors, and then estimating those parameter values using the rest of a set of 20,000 simulations. **A:** v (Model bias). **B:** p_{att} (Content bias). **C:** α (Conformist bias). **D:** μ (Mutation rate).

A

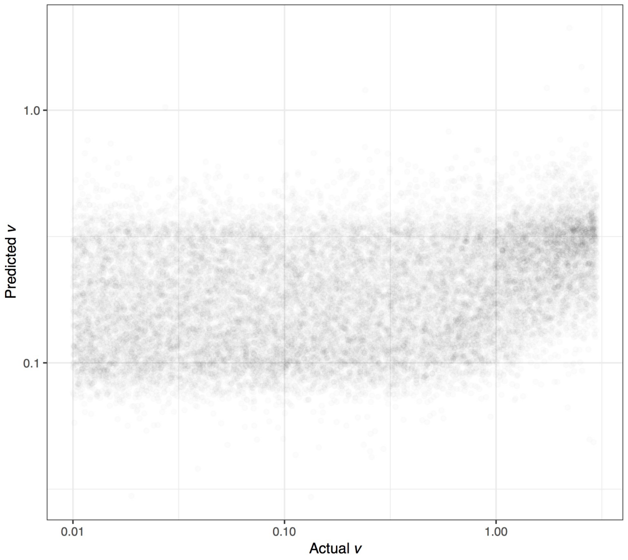

B

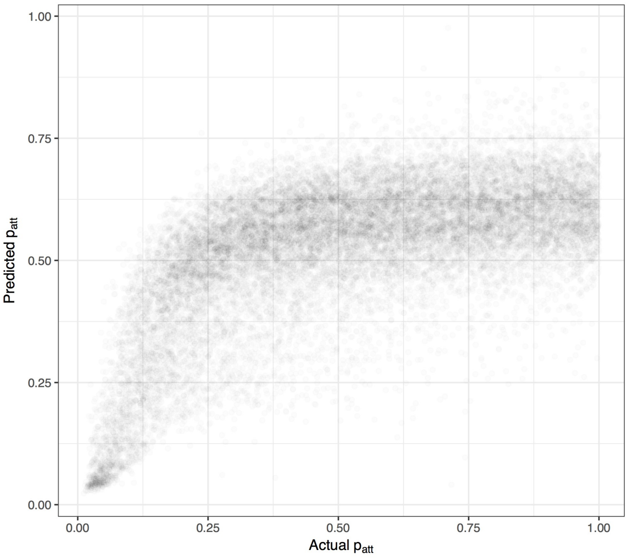

C

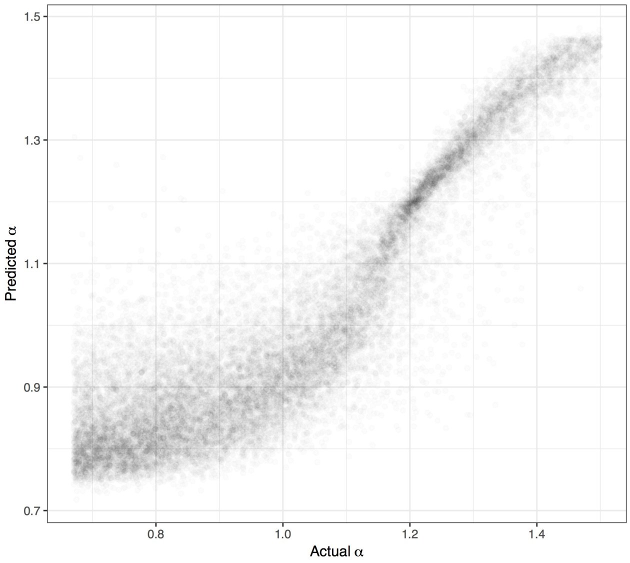

D

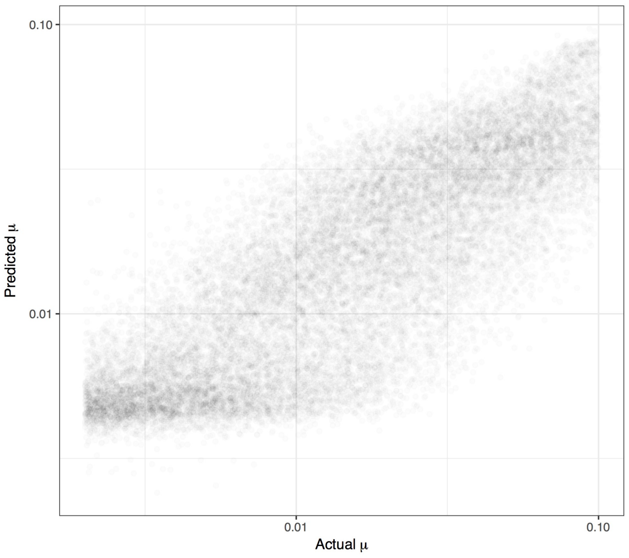

Supplementary Fig. 7 Identifiability of mutation rate in the face of varying population sizes. Figures show the ratio between mutation rates, and the ratio between population sizes in two simulations. Plotted are pairs of simulations that showed a close match in their PLS dimensions. **A:** with no conformist bias, and showing the clear negative correlation predicted by classical population genetics. **B:** with α=1.33 (conformist bias matching our median estimate from our ABC analysis), showing no trade-off and with values of μ-ratio clustering around 1 (suggesting a good ability to estimate mutation rate).

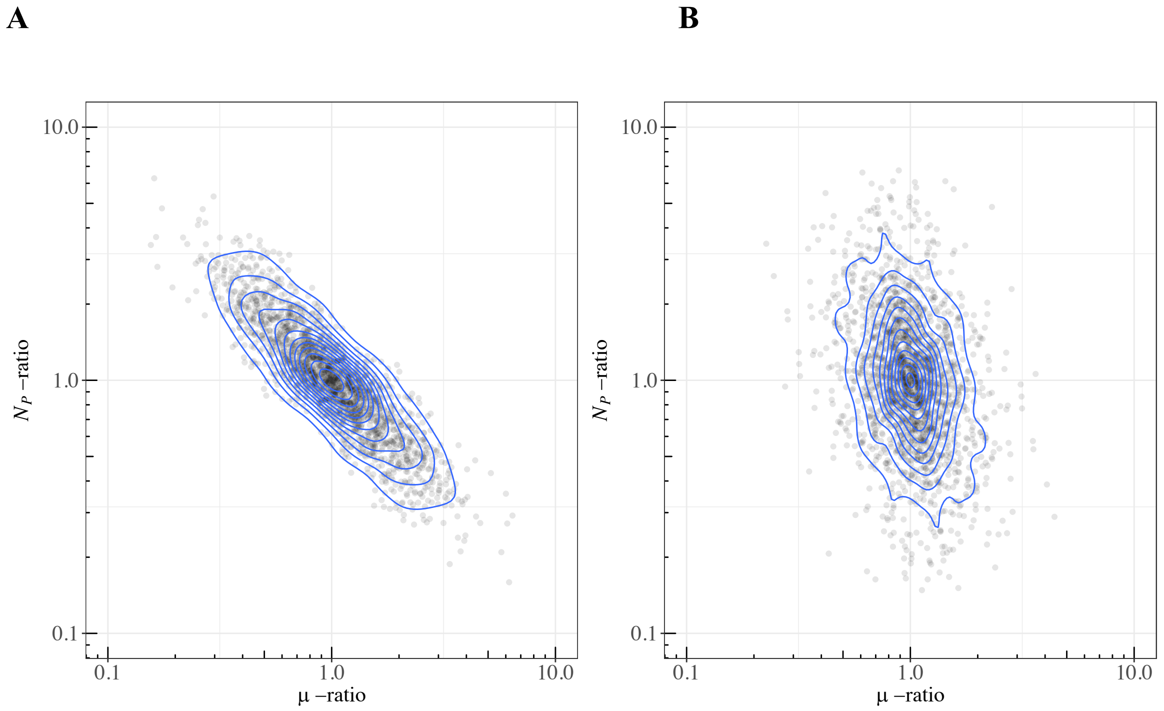

Supplement: Supplementary file 1 — Supplementary Information [file 41467_2018_4728_MOESM1_ESM.pdf]
